# Editorialets

**Published:** 1907-11

**Authors:** 


### Editorialets.

DR. S. E. HUDSON, of Austin, is taking a p.-g. course in Berlin.

AND GEORGIA! And now Grayson county, Texas, has abolished the saloon.

ETHICS OF THE "NEW DISPENSATION," *a la* Octopus: Strain at a "proprietary" and swallow an Osteopath.

TAINTED MONEY.—Revenue from whisky license. Tainted with the blood of men and the tears of women and children.

CIVILIZATION IS ADVANCING.—Oklahoma, so recently an Indian nation, has a constitution in which prohibition is incorporated.

OPSONINS AND BACTERIAL VACCINES.—By Dr. E. M. Houghton, with directions for determining the opsonic index of the blood by Dr. E. C. L. Miller. This is a neat pamphlet reprint from the *Therapeutic Gazette* of January 15th and March 15th. A copy may be had upon request, naming the "Red Back." Send a postal card to Parke, Davis & Co., Detroit, Mich.

---

It Is Exceedingly Gratifying to see the *American Journal of Clinical Medicine* come out strong editorially for the abolition of the accursed saloon.

---

Dr. Daniel: Congratulations on your October number. It is one of the best you have ever put out.

The Abbott Alkaloidal Co.

---

The Saloon, the Modern Inferno.—"Who enters here leaves hope behind,"—and health and happiness and self-respect and fortune and friends, and fixes manacles upon his unborn children, dooming them to disease and sorrow and early death. The moderate drinker of today is the drunkard of tomorrow, and his children and their children fill the asylums and prisons.

---

The Manor Sanitarium.—A commodious and well furnished and equipped building, with ample grounds and attractive surroundings, is offered for lease or sale. Correspondence solicited.

A. K. Anderson,
Jno. E. Hill,
John E. Sellstrom,
For Executive Board.

---

Of Two Evils Choose—Neither.—The canteen or the low whisky dives that spring up around army posts? All the army officers say the canteen should be restored; that by reason of saloons accessible to the men insubordination, desertion, drunkenness and disease have increased. What's the matter with *abolishing the saloon,* as well as the canteen? True, the canteen is the lesser evil, but why should there be either? The *saloon must go.*

---

Dr. Josephine Kingsley died at her home in San Antonio, Texas, October 14th (ult.), aged 63. Dr. Kingsley was a sister and associate in practice of Dr. B. F. Kingsley. This firm was amongst the most eminent, popular and distinguished in Texas, and Dr. Kingsley's death will be mourned by a wide circle of admiring and attached friends. It is a distinct loss to the profession and the State. Dr. Kingsley was eminent and distinguished no less in social circles, and was ever forward in charitable work. She was also a contributor at intervals to the current literature of medicine, and was the author of several valuable papers contributed to the State Medical Association and which grace the annual volumes of the Transactions.

---

A THING OF BEAUTY AND A JOY FOREVER.—I mean the Duplex phonograph. I have one and am simply delighted with it. It is perfection. It is a source of no end of pleasure and entertainment, and is very ornamental. Clear, soft or loud, at your will. It furnishes dance music in the parlor or band music for your gallery. The Duplex is not in the combine, and you save 70 per cent and get the *best in the market.* You will be asked $75 to $100 for a Victor or Columbia not near so large, showy or good as the Duplex, which is delivered free for $29.85, and you can try it a week before you pay a cent and get three records and three hundred needles free. Get one for Christmas for all the family, and take my word for it—they and you will be delighted. A postal card gets booklet free. Mention this.

